# A phase I dose-escalation study of LY2875358, a bivalent MET antibody, given as monotherapy or in combination with erlotinib or gefitinib in Japanese patients with advanced malignancies

**DOI:** 10.1007/s10637-016-0370-7

**Published:** 2016-09-01

**Authors:** Kiyotaka Yoh, Toshihiko Doi, Hironobu Ohmatsu, Takashi Kojima, Hideaki Takahashi, Yoshitaka Zenke, Volker Wacheck, Sotaro Enatsu, Takashi Nakamura, Kellie Turner, Kazunori Uenaka

**Affiliations:** 1 National Cancer Center Hospital East, 5-1, Kashiwanoha 6-chome, Kashiwa, Chiba 277-8577 Japan; 2 Eli Lilly and Company, Indianapolis, IN USA; 3 Eli Lilly Japan K. K., Kobe, Japan

**Keywords:** Antibodies, monoclonal, Epidermal growth factor receptor, LY2875358, MET, Pharmacokinetics, Solid tumors

## Abstract

*Background* MET is a tyrosine kinase receptor involved in the regulation of cell proliferation and migration. Reported here are the phase I dose-escalation results for LY2875358, a monoclonal antibody against MET, in Japanese patients with advanced malignancies. *Methods* The study comprised a 3 + 3 dose-escalation part for LY2875358 monotherapy in patients with advanced malignancies (Part A) followed by an assessment of LY2875358 in combination with erlotinib or gefitinib in patients with non-small cell lung cancer (Part B). LY2875358 was administered once every 2 weeks. The primary objective was to evaluate the safety and tolerability of LY2875358; secondary objectives included evaluation of pharmacokinetics, pharmacodynamics, and antitumor activity. *Results* Eleven patients received LY2875358 monotherapy at 3 dose levels (700 mg, *N* = 3; 1400 mg, *N* = 3; 2000 mg, *N* = 5) and 6 patients received LY2875358 2000 mg in combination with erlotinib (*N* = 3) or gefitinib (*N* = 3). No dose-limiting toxicities or serious adverse events related to LY2875358 were observed. The most frequently reported drug-related adverse events were hypoalbuminemia (2 patients) in Part A and dermatitis acneiform (4 patients) in Part B. LY2875358 area under the curve (AUC) and maximum concentration (C_max_) increased with dose over the dose range of 700 mg to 2000 mg. A best response of stable disease was achieved by 2/11 patients in Part A and 4/6 patients in Part B (disease control rate: 35 %). *Conclusions* LY2875358 at doses up to 2000 mg demonstrated a favorable safety and tolerability profile as monotherapy or in combination with erlotinib or gefitinib in Japanese patients with advanced malignancies.

## Introduction

The hepatocyte growth factor (HGF) receptor, also known as MET, is a tyrosine kinase receptor that has been associated with tumor progression and metastasis [1–3]. Aberrant activation of the HGF/MET signaling pathway promotes tumor cell growth, survival, angiogenesis, invasion, and metastasis [1–3]. Many tumor types, including gastric, colorectal, renal, breast, pancreatic, lung, thyroid, and hepatocellular carcinoma, have aberrant activation of the HGF/MET signaling pathway [1, 4–8]. Activation of this pathway can occur by several mechanisms, including ligand-dependent activation by HGF and ligand-independent, constitutive activation of MET through gene amplification or genetic mutations [3, 4, 9]. Elevated HGF expression and *c-MET* amplification have been linked with acquired resistance to epidermal growth factor receptor tyrosine kinase inhibitors (EGFR TKIs) in patients with non-small cell lung cancer (NSCLC) [10–13]. As the HGF/MET signaling pathway plays a role in several key processes underlying tumor progression, targeting this pathway is considered a promising therapeutic strategy for the treatment of patients with MET-expressing cancers, including those with NSCLC who have acquired resistance to EGFR TKIs.

LY2875358 is a humanized, bivalent, monoclonal, immunoglobulin G4 (IgG4) antibody against MET [14]. It prevents ligand-dependent and ligand-independent activation of the MET/HGF pathway; by binding to MET, LY2875358 blocks the binding of HGF to MET and thereby inhibits HGF ligand-dependent induction of MET phosphorylation [14]. In addition, binding of LY2875358 to MET results in internalization and degradation of MET, leading to suppression of ligand-independent cell proliferation and tumor growth in preclinical models where MET is constitutively activated [14]. These characteristics suggest that LY2875358 is active against tumors whether they are driven by elevated HGF expression or constitutive MET activation. The first human dose study of LY2875358 showed that administration as monotherapy (dose range: 20 mg to 2000 mg) or in combination with erlotinib (dose range: 700 mg to 2000 mg) was well tolerated in patients with advanced solid tumors [15]. No dose-limiting toxicities (DLTs), serious adverse events (SAEs), or ≥Grade 3 adverse events (AEs) possibly related to LY2875358 were observed for LY2875358 monotherapy or LY2875358 plus erlotinib in this mainly Caucasian patient population. The recommended phase II dose (RPTD) range of LY2875358 was determined to be 700 mg to 2000 mg intravenously every 2 weeks for both monotherapy and combination therapy with erlotinib.

The aim of this phase I study was to investigate the safety of LY2875358 in Japanese patients. LY2875358 was administered as monotherapy in patients with advanced solid tumors (Part A) or in combination with erlotinib or gefitinib in patients with advanced NSCLC (Part B). The primary objective of the study was to assess the safety and tolerability of LY2875358 at doses up to and including the RPTD range from the first human dose study (Study JTBA) [15]. Secondary objectives included the assessment of toxicity, pharmacokinetics, antitumor activity, and biomarker analysis.

## Materials and methods

### Study design

This study (Study JTBD) was a phase I, single-center, open-label, nonrandomized, dose-escalation study of LY2875358 in Japanese patients with advanced and/or metastatic malignancies. The study consisted of two parts: a dose-escalation part for LY2875358 monotherapy (Part A) followed by a cohort-expansion part for LY287538 in combination with erlotinib (Part B1) or gefitinib (Part B2). Dose escalations of LY2875358 were performed following a standard 3 + 3 design. The study protocol was approved by the site’s ethics review board and conducted in accordance with the Declaration of Helsinki and Good Clinical Practice guidelines. All patients provided written informed consent before undergoing any study procedure. Patients who continued study treatment after the first cycle of treatment signed a second informed consent form before starting the second cycle of treatment. The study was registered at www.clinicaltrials.gov (NCT01602289).

### Study population

Patients with histological or cytological evidence of advanced and/or metastatic malignancies who were considered an appropriate candidate for experimental therapy after use of standard therapies were eligible for LY2875358 monotherapy (Part A). For the LY2875358 combination cohorts (Part B), patients were eligible if they had histological or cytological evidence of Stage IV NSCLC [16] (with activating EGFR mutations for Part B2), had no other effective therapeutic option, and were suitable for erlotinib (Part B1) or gefitinib (Part B2) therapy per the Japanese package insert [17, 18]. Previous EGFR TKI treatment was permitted.

Additional inclusion criteria for the study included: age ≥ 20 years; measurable or unmeasurable disease by Response Evaluation Criteria in Solid Tumors (RECIST) version 1.1 [19]; Eastern Cooperative Oncology Group (ECOG) performance status (PS) of ≤2; and adequate organ function. Exclusion criteria included: symptomatic central nervous system malignancy or metastasis (Part A); brain metastases that were symptomatic or required ongoing treatment with steroids or radiation therapy (Part B); active infection, including human immunodeficiency virus and hepatitis A, B, and C; previous treatment with any MET targeting agent; receiving warfarin therapy (Part B); concomitant treatment with cytochrome P450 3A4 modulators (no treatment with a modulator within 14 days of Cycle 1, Day 1; Part B); evidence of clinically active interstitial lung disease or pre-existing interstitial lung disease risk as demonstrated by computed tomography (Part B); and any disease of acute lung injury, idiopathic pulmonary fibrosis, pneumonitis, or pneumoconiosis (Part B).

### Study treatments

Patients in the dose-escalation part (Part A) were enrolled sequentially into LY2875358 dose cohorts of 700 mg, 1400 mg, and 2000 mg using a flat dosing scheme. Patients were administered LY2875358 intravenously over approximately 90 min (700 mg cohort) or 150 min (1400 mg and 2000 mg cohorts) on Day 1 and Day 15 of a 28-day cycle, until any discontinuation criterion was met.

In Part B, patients with Stage IV NSCLC were administered LY2875358 2000 mg, using the same schedule and method in Part A, in combination with erlotinib administered orally as a once-daily dose of 150 mg (Part B1) or gefitinib administered orally as a once-daily dose of 250 mg (Part B2).

### Dose-escalation method

Dose escalation of LY2875358 in Part A occurred sequentially over the doses of 700 mg, 1400 mg, and 2000 mg until the criteria for reaching the maximum tolerated dose (MTD) were met or the highest planned dose cohort was completed. The MTD was defined as the highest tested dose that had <33 % probability of causing a DLT. A DLT was defined as an AE during the DLT evaluation period (the first cycle: 28 days) that was possibly related to the study drug (LY2875358 alone or in combination with erlotinib/gefitinib) and fulfilled one of the following criteria using the National Cancer Institute (NCI) Common Terminology Criteria for Adverse Events (CTCAE) version 4.02: ≥Grade 3 nonhematological toxicity; Grade 4 neutropenia of duration >7 days; febrile neutropenia; or Grade 3 thrombocytopenia with ≥Grade 2 bleeding or Grade 4 thrombocytopenia with or without bleeding, regardless of duration.

### Dose adjustments and delays

Dose adjustments of LY2875358 were not permitted during the study. Before the start of each administration of LY2875358, hematological toxicities (except anemia) and nonhematological toxicities (except alopecia, fatigue, skin rash, nausea, vomiting, or diarrhea that could be controlled with treatment) possibly related to LY2875358 had to resolve to ≤Grade 1 or baseline. The start of each administration of LY2875358 could be delayed for up to 2 weeks to allow sufficient time for recovery.

The erlotinib dose could be reduced for toxicities attributable to erlotinib at the discretion of the investigator and according to the Japanese package insert [17]. For gefitinib, poorly tolerated diarrhea, treatment-related AEs of the skin, or any other AE that the investigator believed was attributable to gefitinib could be managed by dosing interruption (up to 14 days), followed by reinstatement of the 250 mg daily dose at the discretion of the investigator and according to the Japanese package insert [18].

### Clinical assessments

Adverse events were graded using the NCI CTCAE version 4.02. Tumor response was assessed by RECIST version 1.1 [19] every other cycle after Cycle 2 or as clinically indicated. Responses required confirmation by the same tumor imaging method within 4 weeks of the initial observation of an objective response. The objective response rate (ORR) was defined as the percentage of patients who experienced a complete response (CR) or partial response (PR). The disease control rate (DCR) was defined as the percentage of patients who experienced a CR, PR, or stable disease (SD). Progression-free survival (PFS) was measured from the date of the first dose of study drug to the first date of objective progression of disease or the date of death from any cause, whichever occurred first.

### Dose intensity

Relative dose intensity was the actual dose intensity (in mg/week) expressed as a percentage of the planned weekly dose.

### Pharmacokinetics

Blood samples were collected for pharmacokinetic analysis during Cycle 1 as follows: before the infusion on Day 1, mid-infusion, at the end of the infusion, 2, 4, 6, 8, and 24 h (Day 2) after the end of the infusion, Day 4–6 at any time, Day 8, before the infusion on Day 15, at the end of the infusion, 2, 4, and 6 h after the end of the infusion, and Day 22. Blood samples were collected for pharmacokinetic analysis during Cycle 2 as follows: before the infusion on Day 1, mid-infusion, at the end of the infusion, 2 and 4 h after the end of the infusion, Day 8, before the infusion on Day 15, and at the end of the infusion. The pharmacokinetic parameters for LY2875358, erlotinib, and gefitinib were computed by standard noncompartmental methods using Phoenix WinNonlin 6.3 software (Certara USA, Inc., Princeton, NJ, USA).

### Biomarker analysis

Immunohistochemical staining using the anti-MET antibody A2H2-3 [20] was conducted for optionally available archival tumor samples. A composite scoring system was devised to determine the status of MET by immunohistochemistry. Tumor samples with ≥50 % of cells stained 2+ or 3+ for MET expression were considered MET diagnostic positive. The H-score was calculated as described previously [21] as the sum of 1 × (% of 1+ cells) + 2 × (% of 2+ cells) + 3 × (% of 3+ cells). Fluorescence in situ hybridization for the *c-MET* gene was conducted to assess amplification in tumor samples. The *c-MET* gene was considered to be amplified if the ratio of the average copy number per cell of *c-MET* to the average copy number per cell of the control chromosome 7 centromere (CEP7) was ≥2. Serum was collected pre-dose and throughout the study and assessed for the circulating extracellular domain (ECD) of MET by a validated enzyme-linked immunosorbent assay.

### Statistical analysis

The sample size was determined by the incidence of DLTs (up to 6 patients per cohort, up to 30 patients in total). All patients who received at least 1 dose of any study drug were evaluated for safety, toxicity, and antitumor activity (full analysis set). All patients who met the DLT criteria at a particular dose level were evaluated for DLTs (DLT evaluation set). Descriptive statistics were used to summarize safety and antitumor activity. Analyses were performed using SAS 9.2 (SAS Institute, Cary, NC, USA).

## Results

### Patient disposition

A total of 17 patients were enrolled in Study JTBD and received at least 1 dose of LY2875358. Eleven patients were enrolled in the LY2875358 monotherapy cohorts and received 700 mg (*N* = 3), 1400 mg (*N* = 3), or 2000 mg (*N* = 5) LY2875358. Two patients in the 2000 mg monotherapy cohort were not evaluable for DLTs and were replaced (1 patient discontinued study treatment after the first dose of study drug because of an AE [hypoalbuminemia] not related to study drug; 1 patient discontinued study treatment after the first dose of study drug because of progressive disease [PD]). After completion of Part A, a total of 6 patients were enrolled in Part B to receive LY2875358 in combination with erlotinib (Part B1; *N* = 3) or gefitinib (Part B2; *N* = 3). All patients enrolled in the study were included in the safety and pharmacokinetic analyses.

### Demographic and baseline clinical characteristics

All patients in this study had Stage IV solid tumors and had received prior systemic therapy (Table 1). Patients enrolled in Part A had a diagnosis of pancreatic adenocarcinoma, esophageal carcinoma (*n* = 2 for both), gastric adenocarcinoma, biliary tract carcinoma, gall bladder carcinoma, neuroendocrine carcinoma, rectal carcinoma, thymic carcinoma, or gastrointestinal stromal tumors (*n* = 1 for all). All 6 patients in Part B had a diagnosis of lung adenocarcinoma and had received treatment with at least one first-generation EGFR TKI before enrolling in the study. The baseline patient and disease characteristics are summarized in Table 1.

**Table 1:** Patient demographics and baseline disease characteristics

Characteristic	Part A					Part B		
	*n*			*n* (%)		*n*		*n* (%)
	LY2875358 700 mg (*n* = 3)	LY2875358 1400 mg (*n* = 3)	LY2875358 2000 mg (*n* = 5)	Total (*N* = 11)		Part B1 LY2875358 2000 mg + Erlotinib (*n* = 3)	Part B2 LY2875358 2000 mg + Gefitinib (*n* = 3)	Total (*N* = 6)
Sex								
Male	1	1	4	6 (54.5)		1	2	3 (50.0)
Female	2	2	1	5 (45.5)		2	1	3 (50.0)
Age, years								
Mean (min–max)	53.6 (39.0–68.0)	61.6 (56.2–64.3)	64.0 (52.8–75.4)	60.5 (39.0–75.4)		64.3 (56.1–75.0)	61.1 (51.4–73.4)	62.7 (51.4–75.0)
Age group								
<65 years	2	3	3	8 (72.7)		2	2	4 (66.7)
≥65 years	1	0	2	3 (27.3)		1	1	2 (33.3)
Prior systemic therapy	3	3	5	11 (100)		3	3	6 (100)
Prior surgery	2	2	3	7 (63.6)		0	1	1 (16.7)
Prior radiotherapy	2	1	0	3 (27.3)		1	2	3 (50.0)
ECOG PS								
0	2	2	5	9 (81.8)		2	1	3 (50.0)
1	1	1	0	2 (18.2)		1	2	3 (50.0)

Abbreviations: *ECOG* Eastern Cooperative Oncology Group, *min* minimum, *max* maximum, *PS* performance status

### Safety and tolerability

LY2875358 treatment was well tolerated, both when administered as monotherapy and when administered in combination with erlotinib or gefitinib. No DLTs were observed in any patient enrolled in this study.

Six of 11 patients (54.5 %) in Part A reported at least 1 AE possibly related to study drug (Table 2). The most frequently reported AE considered possibly related to LY2875358 was hypoalbuminemia, which was reported by 2 patients (1 patient each in the 700 mg and 2000 mg cohorts). Three patients reported 7 Grade ≥ 3 AEs; of these, the Grade 3 anemia reported by 1 patient in the 700 mg cohort was considered possibly related to LY2875358. This patient (who had Grade 1 anemia at baseline) reported Grade 2 anemia on Day 36 of Cycle 1, which had developed to Grade 3 at the time of treatment discontinuation. A total of 3 SAEs in 2 patients were reported in Part A, none of which were considered possibly related to LY2875358. This included 1 patient in the 1400 mg cohort with Grade 4 sepsis and Grade 3 fatigue, and 1 patient in the 2000 mg cohort with Grade 4 depressed level of consciousness. One patient in the 1400 mg cohort reported a Grade 2 infusion-related reaction considered possibly related to LY2875358.

**Table 2:**
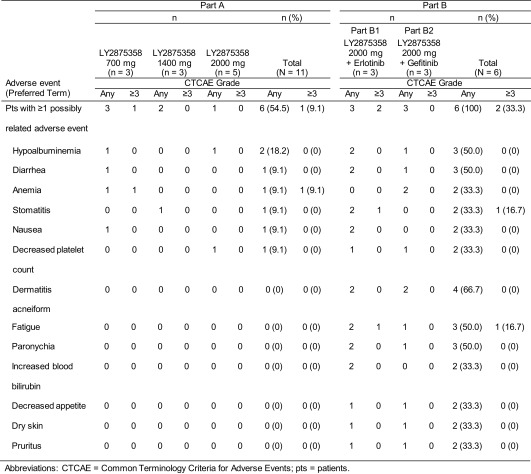
Most frequently occurring adverse events possibly related to study drug

All patients in Part B receiving LY2875358 treatment in combination with erlotinib or gefitinib reported at least 1 AE possibly related to study drugs (Table 2). The most frequently reported AEs considered possibly drug related (LY2875358 or erlotinib/gefitinib) included dermatitis acneiform (*n* = 4), hypoalbuminemia (*n* = 3), diarrhea (*n* = 3), fatigue (*n* = 3), and paronychia (*n* = 3). One patient in Part B2 experienced peripheral edema considered possibly related to LY2875358. Two patients in Part B reported ≥Grade 3 AEs, both of which (Grade 3 fatigue, Grade 3 stomatitis) were possibly related to LY2875358. One patient in Part B1 reported an SAE of Grade 3 metastases to the thoracic spine, which was not considered related to study drug.

Progressive disease was the primary reason for discontinuation from study treatment (9/11 patients [81.8 %] in Part A and 3/6 patients [50.0 %] in Part B). Two patients discontinued study treatment because of an AE possibly related to LY2875358: 1 patient in the 700 mg cohort because of Grade 2 hypoalbuminemia and 1 patient in the LY2875358 plus gefitinib cohort because of a Grade 2 decreased platelet count. No AEs leading to death were reported in this study.

### Pharmacokinetics

**Fig. 1:**
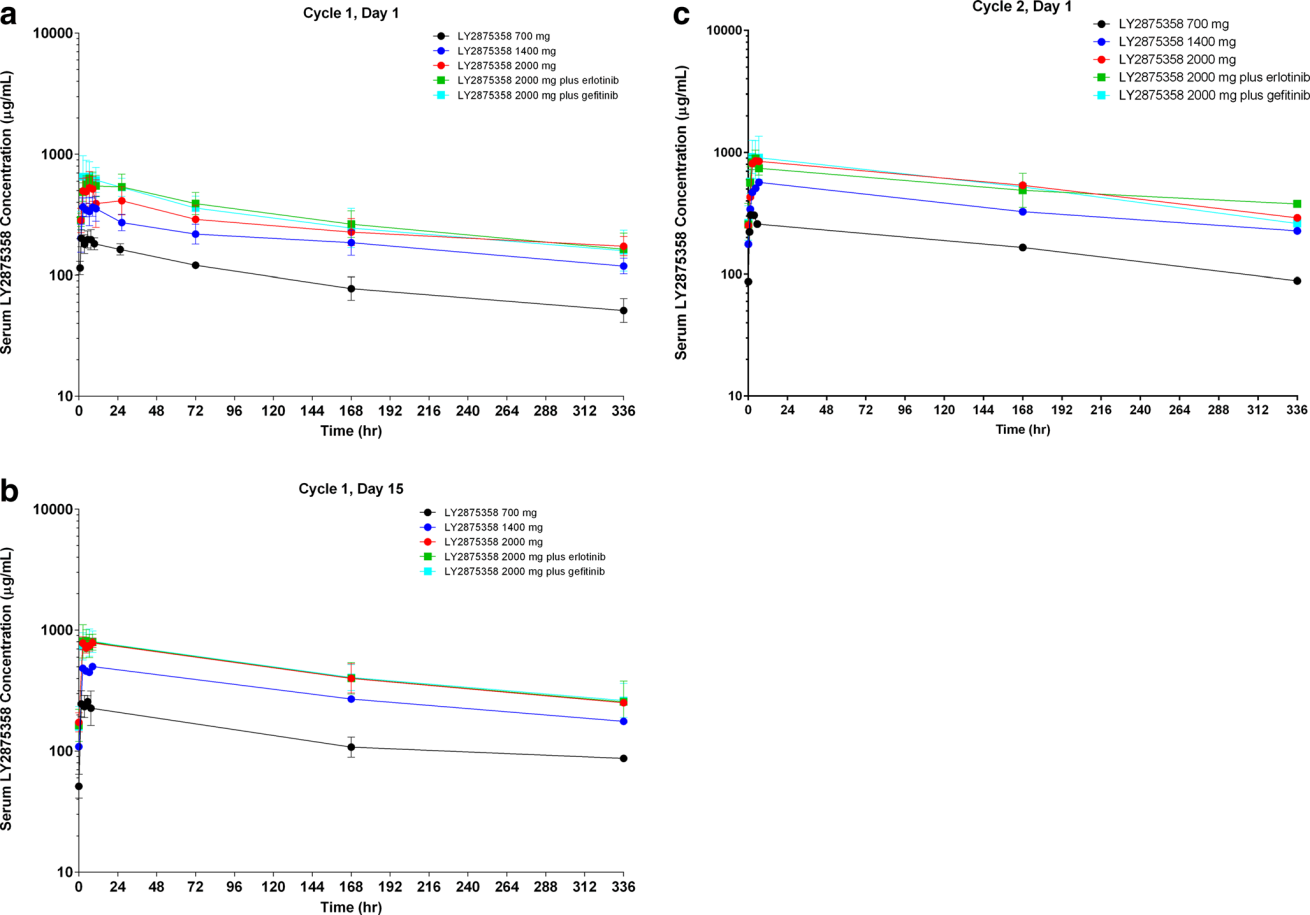
Semi-logarithmic plot of mean serum LY2875358 concentration-time profiles following intravenous infusion of LY2875358 700 mg, 1400 mg, 2000 mg, 2000 mg plus erlotinib 150 mg/day, or 2000 mg plus gefitinib 250 mg/day on **a** Day 1 of Cycle 1, **b** Day 15 of Cycle 1, and **c** Day 1 of Cycle 2

The pharmacokinetic parameters of LY2875358 when administered as monotherapy or in combination with erlotinib or gefitinib are shown in Table 3. Following administration of LY2875358 monotherapy on Day 1 of Cycle 1 in Part A, the maximum concentration (C_max_) and area under the concentration time-curve (AUC) from 0 h to infinity (AUC(0-∞)) increased with increasing dose of LY2875358, whereas clearance was similar across doses (Table 3). The t_max_ (time at which maximum concentration is reached) values for the patients in the 3 LY2875358 monotherapy cohorts ranged from 1.47 h to 10.53 h (Table 3). The mean plasma LY2875358 concentration-time profiles following administration of LY2875358 on Day 1 of Cycle 1, Day 15 of Cycle 1, and Day 1 of Cycle 2 for the 3 LY2875358 monotherapy cohorts are shown in Fig. 1. The geometric mean accumulation ratio of LY2875358 on Day 1 of Cycle 2 based on the AUC during one dosing interval (AUC(0-τ)) was within the range of 1.5 to 2.0 for the 3 LY2875358 monotherapy cohorts. The geometric mean trough concentration of LY2875358 on Day 1 of Cycle 2 was 87.1, 176, and 251 μg/mL in the 700 mg, 1400 mg, and 2000 mg dose cohorts, respectively.

**Table 3:** Summary of pharmacokinetic parameters following administration of LY2875358 on Day 1 of Cycle 1

	Geometric mean (CV%)				
	Part A			Part B	
Parameter	LY2875358 700 mg (*n* = 3)	LY2875358 1400 mg (*n* = 3)	LY2875358 2000 mg (*n* = 5)	Part B1 LY2875358 2000 mg + Erlotinib (*n* = 3)	Part B2 LY2875358 2000 mg + Gefitinib (*n* = 3)
C_max_, μg/mL	219 (17)	395 (20)	575 (26)	589 (21)	704 (34)
t_max_^a^, hr	1.50 (1.47–7.52)	6.50 (4.48–10.53)	6.53 (2.58–8.50)	6.48 (4.50–8.53)	8.38 (2.55–10.63)
t_1/2_^b^, hr	198 (157–265)	290 (266–342)	318 (262–637)	204 (163–238)	194 (137–288)
AUC(0-t_last_), μg•hr./mL	30,900 (15)	63,000 (22)	96,400 (24)	98,500 (27)	92,600 (36)
AUC(0-∞), μg•hr./mL	44,900 (28)	113,000 (15)	154,000 (35)	145,000 (35)	134,000 (56)
CL, L/h	0.0158 (28)	0.0125 (15)	0.0131 (36)	0.0139 (35)	0.0150 (56)
V_ss_, L	4.45 (4)	5.14 (27)	5.85 (33)	4.03 (20)	4.14 (17)

Abbreviations: *AUC(0-t*_*last*_ *)* area under the concentration-time curve from 0 h to the time of the last measurable concentration, *AUC(0-∞)* area under the concentration-time curve from 0 h to infinity, *CL* clearance, *C*_*max*_ maximum concentration, *CV* coefficient of variation, *t*_*1/2*_ half-life, *t*_*max*_ time at which maximum concentration is reached, *V*_*ss*_ volume of distribution at steady state

^a^Median (range)

^b^Geometric mean (range)

The pharmacokinetics of LY2875358 were not obviously affected by the administration of erlotinib or gefitinib in Part B1 or B2, respectively (Table 3, Fig. 1). In particular, C_max_, AUC(0-∞), and clearance were similar between the LY2875358 2000 mg dose cohort and the LY2875358 2000 mg plus erlotinib and LY2875358 2000 mg plus gefitinib cohorts (Table 3). The t_max_ values for the patients in the LY2875358 2000 mg plus erlotinib and LY2875358 2000 mg plus gefitinib cohorts ranged from 2.55 h to 10.63 h (Table 3). The mean plasma LY2875358 concentration-time profiles of LY2875358 2000 mg plus erlotinib and LY2875358 2000 mg plus gefitinib were similar to that of LY2875358 2000 mg monotherapy following administration of LY2875358 on Day 1 of Cycle 1, Day 15 of Cycle 1, and Day 1 of Cycle 2 (Fig. 1). The geometric mean accumulation ratio of LY2875358 when administered in combination with erlotinib or gefitinib was within the range of 1.5 to 2.0, as observed when LY2875358 was administered as monotherapy. The geometric mean trough concentration of LY2875358 on Day 1 of Cycle 2 was 233 and 251 μg/mL in the LY2875358 2000 mg plus erlotinib and LY2875358 2000 mg plus gefitinib cohorts, respectively.

### Dose intensity of LY2875358, erlotinib, and gefitinib

In Part A, patients received 1 to 5 cycles of LY2875358 treatment, with a mean relative dose intensity of LY2875358 of 100 %, 98.8 %, and 79.1 % in the 700 mg, 1400 mg, and 2000 mg cohorts, respectively. Interruption of an LY2875358 infusion occurred in 1 patient in the 1400 mg cohort because of a Grade 2 infusion-related reaction; the interruption lasted approximately 3 h, with the patient completing the full dose after the interruption.

In Part B, patients received 2 to 6 cycles of treatment. The mean relative dose intensity of LY2875358 and erlotinib in Part B1 was 87.9 % and 99.4 %, respectively. Omission of an erlotinib dose occurred for 1 patient because of an AE (fatigue). The mean relative dose intensity of LY2875358 and gefitinib in Part B2 was 89.8 % and 99.8 %, respectively. Omission of a gefitinib dose occurred for 1 patient because of an investigator decision. No patients had a dose reduction for erlotinib or gefitinib.

### Antitumor activity

No complete or partial responses were observed in this study. In Part A, 2/11 patients had a best response of SD (Table 4), for a DCR of 18.2 %. This included 1 patient with thymic carcinoma who had PFS of 43+ days (censored value) and 1 patient with gall bladder carcinoma who had PFS of 139 days. In Part B, 4/6 patients had a best response of SD (*n* = 2 in each part, Table 4), for a DCR of 66.7 %, and PFS ranged from 50 to 174 days.

**Table 4:** Antitumor activity of LY2875358 as monotherapy and in combination with erlotinib or gefitinib

Cohort	Patient number	Pathological diagnosis	ECOG PS^a^	No. of prior regimens^b^	No. of cycles	BOR	PFS, days	Change from baseline^c^, cm (%)	MET IHC					
									0+, %	1+, %	2+, %	3+, %	H-score	MET/CEP7 ratio
*Part A*														
700 mg	1101	Gastrointestinal stromal tumors	0	4	2	PD	57	8.5 (50.0)	100	0	0	0	0	1.16
700 mg	1102	Esophageal carcinoma	1	4	1	PD	29	1.5 (18.1)	50	40	10	0	60	0.92
700 mg	1103	Thymic carcinoma	0	2	1	SD	43+	0.3 (6.5)	100	0	0	0	0	NA
1400 mg	1104	Pancreatic adenocarcinoma	0	5	2	PD	58	1.9 (37.3)	20	10	70	0	150	0.88
1400 mg	1105	Esophageal carcinoma	0	3	2	PD	48	2.0 (31.7)	NA	NA	NA	NA	NA	NA
1400 mg	1106	Pancreatic adenocarcinoma	1	2	1	PD	23	3.6 (36.7)	NA	NA	NA	NA	NA	NA
2000 mg	1107	Gall bladder carcinoma	0	2	5	SD	139	0.1 (1.4)	70	20	10	0	40	1.01
2000 mg	1108	Neuroendocrine carcinoma	0	3	2	PD	52	0.5 (11.6)	NA	NA	NA	NA	NA	NA
2000 mg	1109	Biliary tract carcinoma	0	3	1	PD	29+	7.3 (52.1)	NA	NA	NA	NA	NA	NA
2000 mg	1111	Gastric adenocarcinoma	0	3	1	PD	14	1.5 (19.0)	NA	NA	NA	NA	NA	NA
2000 mg	1112	Rectal carcinoma	0	2	1	PD	29	1.4 (15.7)	NA	NA	NA	NA	NA	NA
*Part B1*														
LY2875358 + erlotinib	1121	Lung adenocarcinoma	1	7	2	SD	58	−0.6 (−20.0)	NA	NA	NA	NA	NA	NA
LY2875358 + erlotinib	1122	Lung adenocarcinoma	0	4	2	SD	57+	0 (0)	70	20	10	0	40	0.95
LY2875358 + erlotinib	1124	Lung adenocarcinoma	0	10	2	PD	50	1.2 (17.6)	NA	NA	NA	NA	NA	NA
*Part B2*														
LY2875358 + gefitinib	1131^d^	Lung adenocarcinoma	1	5	6	PD	53	−8.0 (−49.1)	NA	NA	NA	NA	NA	NA
LY2875358 + gefitinib	1132	Lung adenocarcinoma	0	5	2	SD	52+	0 (0)	NA	NA	NA	NA	NA	NA
LY2875358 + gefitinib	1133	Lung adenocarcinoma	1	6	6	SD	174	NA	NA	NA	NA	NA	NA	NA

Abbreviations: *BOR* best overall response, *CEP7* chromosome 7 centromere, *ECOG* Eastern Cooperative Oncology Group, *IHC* immunohistochemistry, *NA* not available, *PD* progressive disease, *PFS* progression-free survival, *PS* performance status, *SD* stable disease, + = censored value

^a^Before Cycle 1

^b^Of systemic therapy

^c^Change in target lesion at the time that BOR was observed

^d^Note that this patient had shrinkage of the target lesion by 49.1 % but developed a new lesion (brain metastases), resulting in a BOR of PD

### Biomarker analyses

MET expression status from archival tumor samples was available for 6 patients, of whom 1 patient (700 mg cohort, pancreatic adenocarcinoma) was MET diagnostic positive, having 70 % of cells staining 2+ for MET expression (Table 4). Amplification status of *c-MET* was evaluable for 5 patients, none of whom had amplification of the *c-MET* gene (Table 4).

**Fig. 2:**
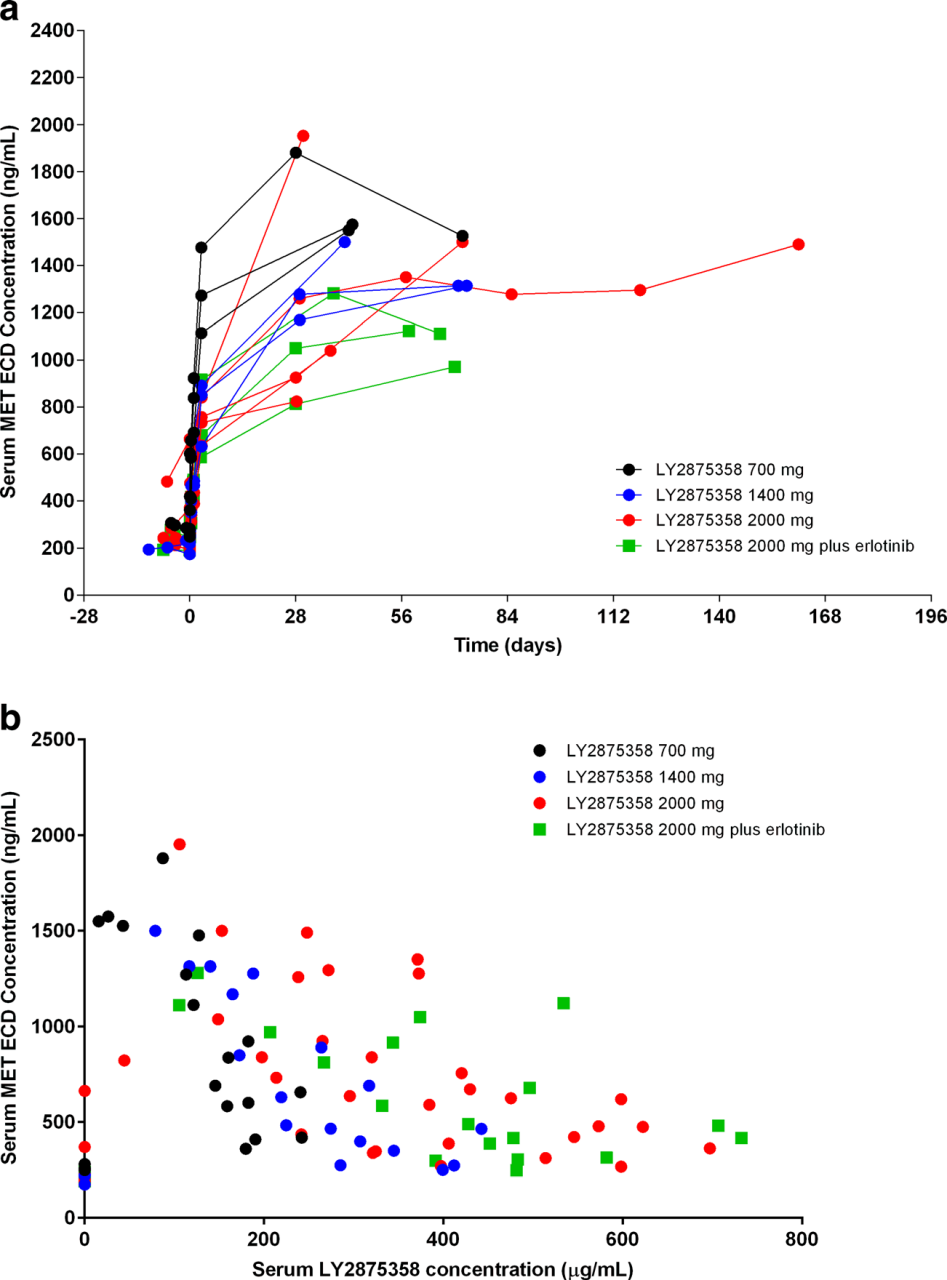
Serum MET ECD concentration vs (**a**) time and (**b**) serum LY2875358 concentration for individual patients in the LY2875358 700 mg, 1400 mg, and 2000 mg cohorts and the LY2875358 2000 mg plus erlotinib cohort. Abbreviations: ECD = extracellular domain

In each of the LY2875358 monotherapy dose cohorts and in the LY2875358 plus erlotinib cohort, an increase in circulating MET ECD levels was observed after initiation of LY2875358 treatment, which plateaued after approximately 4 weeks (Fig. 2a). There did not appear to be a clear dose-dependent relationship between LY2875358 dose and circulating MET ECD levels in the LY2875358 monotherapy dose cohorts or in the LY2875358 plus erlotinib cohort (Fig. 2b). This pharmacodynamic biomarker analysis was not available for the LY2875358 plus gefitinib cohort at the time of the data cut-off.

## Discussion

Here we report the findings from a phase I dose-escalation study for the bivalent MET antibody LY2875358 when administered as monotherapy or in combination with first-generation EGFR TKIs in a Japanese patient population with advanced and/or metastatic malignancies. Besides confirming the safety and tolerability of LY2875358 monotherapy and in combination with erlotinib, this is the first study to evaluate the safety and tolerability of LY2875358 in combination with gefitinib in patients with advanced NSCLC with activating EGFR mutations. Although only a small number of patients received the combination of LY2875358 and gefitinib, the observed safety and tolerability profile appears to be similarly favorable to the combination with erlotinib.

Adverse events possibly related to LY2875358 were observed at a low frequency and were mostly graded mild or moderate in severity in Japanese patients. In both the current study and the previously conducted phase I study of LY2875358, where 23 patients with advanced solid tumors received LY2875358 monotherapy and 14 patients with advanced NSCLC received LY2875358 plus erlotinib [15], there were no DLTs or SAEs possibly related to LY2875358 monotherapy or in combination with erlotinib at any of the LY2875358 doses evaluated. Although no patients in the previous phase I study reported AEs of ≥Grade 3 possibly related to LY2875358 monotherapy [15], 1 patient in the current study (in the 700 mg dose cohort) reported Grade 3 anemia possibly related to LY2875358. There was no dose dependency in the incidence of any AEs possibly related to LY2875358 in the current study. The only possibly related AE reported in more than 1 patient for LY2875358 monotherapy was hypoalbuminemia, which has previously been reported as a class-related effect for agents targeting MET/HGF [22–24]. For patients receiving LY2875358 plus erlotinib, 2 patients in the current study reported Grade 3 AEs (fatigue, stomatitis) possibly related to LY2875358.

For the first time, the combination of LY2875358 and gefitinib was studied in patients with advanced NSCLC. No DLTs, SAEs, or AEs ≥ Grade 3 possibly related to LY2875358 were observed in patients receiving LY2875358 plus gefitinib. The overall safety profile of LY2875358 plus gefitinib appeared to be similar to that observed for LY2875358 plus erlotinib, with mild to moderate hypoalbuminemia, diarrhea, decreased platelet count, dermatitis acneiform, fatigue, paronychia, decreased appetite, dry skin, and pruritus reported for both combinations. The safety profile and the high relative dose intensity of LY2875358 monotherapy and in combination with erlotinib or gefitinib observed in the current study foster the notion that LY2875358 may be safely administered with first-generation EGFR TKIs at doses up to 2000 mg every 2 weeks.

The AUC and C_max_ for LY2875358 increased with dose over the range of 700 mg to 2000 mg in this population of Japanese patients with advanced malignancies and did not appear to be affected by combination with erlotinib or gefitinib. In addition, clearance of LY2875358 was similar in patients receiving LY2875358 2000 mg as monotherapy and in those receiving the same dose of LY2875358 in combination with erlotinib or gefitinib. In general, the t_max_ values observed in this study (1.47 h to 10.63 h) occurred after the end of the LY2875358 infusion (1.5 h for the 700 mg dose and 2.5 h for the 1400 mg and 2000 mg doses), which is consistent with the pharmacokinetics of LY2875358 observed in Caucasian patients in Study JTBA [25]. The doses of LY2875358 used in this study were chosen to achieve exposures associated with tumor growth inhibition in xenograft models. The geometric mean trough concentrations of LY2875358 on Day 1 of Cycle 2, which ranged from 87.1 to 251 μg/mL in the 5 cohorts, exceeded the 60 μg/mL threshold associated with ≥90 % tumor growth inhibition in the MKN45 xenograft model [25].

Although it should be noted that assessment of antitumor activity was not a primary objective of the study and the number of patients evaluated was small, the potential for clinical activity was shown in individual patients. While the majority of patients who received LY2875358 monotherapy discontinued the study with PD, 1 patient with gall bladder cancer in the 2000 mg LY2875358 monotherapy dose cohort received 5 cycles of treatment and had a best response of SD. For LY2875358 in combination with erlotinib or gefitinib, 4 of 6 patients with NSCLC had a best response of SD, including 1 patient in the LY2875358 plus gefitinib group who received 6 cycles of treatment. It should be noted that all 6 patients had been previously treated with at least 1 first-generation EGFR TKI and progressed on this therapy before enrolling in the study.

Circulating MET ECD has been proposed as a surrogate measure of target engagement in studies of MET antibodies [23, 26]. At the doses tested in the current study, MET ECD levels increased from baseline, but there was not a strong dose-dependent relationship between circulating MET ECD and doses of LY2875358. This result is in line with previous reports, including the MET ECD data from the phase I dose-escalation study of onartuzumab, a humanized monovalent monoclonal antibody against MET [23]. In that study, there was a dose-dependent threshold (between 1 mg/kg and 4 mg/kg) for increasing MET ECD levels after onartuzumab treatment, with no further dose-dependent increase in MET ECD with onartuzumab doses higher than 4 mg/kg.

A limitation of the current study is the limited number of patient tumor samples available for assessment of MET expression and *c-MET* amplification, precluding any assessment of correlation between MET expression and antitumor activity of LY2875358. It would be of value to investigate the possibility of such a correlation in future studies of LY2875358, which could inform future patient tailoring strategies to identify patients who might benefit most from LY2875358 treatment.

In conclusion, LY2875358 administered at doses up to 2000 mg every 2 weeks in this phase I study was associated with a favorable safety profile with no DLTs, both when administered as monotherapy and in combination with erlotinib or gefitinib. The tolerability and pharmacokinetic/pharmacodynamic data in Japanese patients with advanced malignancies support the globally recommended phase II dose range of 700 mg to 2000 mg for LY2875358 monotherapy and in combination with erlotinib. LY2875358 is currently being studied in combination with erlotinib in ongoing phase II trials of patients with NSCLC.
